# Glioblastoma Standard of Care: Effects on Tumor Evolution and Reverse Translation in Preclinical Models

**DOI:** 10.3390/cancers16152638

**Published:** 2024-07-24

**Authors:** Louis T. Rodgers, John L. Villano, Anika M. S. Hartz, Björn Bauer

**Affiliations:** 1 Department of Pharmaceutical Sciences, College of Pharmacy, University of Kentucky, Lexington, KY 40536, USA; 2 Markey Cancer Center, College of Medicine, University of Kentucky, Lexington, KY 40536, USA; 3 Department of Medicine, College of Medicine, University of Kentucky, Lexington, KY 40536, USA; 4 Department of Neurology, College of Medicine, University of Kentucky, Lexington, KY 40536, USA; 5 Department of Neurosurgery, College of Medicine, University of Kentucky, Lexington, KY 40536, USA; 6 Sanders-Brown Center on Aging, College of Medicine, University of Kentucky, Lexington, KY 40536, USA; 7 Department of Pharmacology and Nutritional Sciences, College of Medicine, University of Kentucky, Lexington, KY 40536, USA

**Keywords:** glioblastoma, standard of care, resection, radiation therapy, temozolomide, recurrent tumor, reverse translation, preclinical models

## Abstract

**Simple Summary:**

Glioblastoma is the most common and aggressive brain tumor in adults. Despite surgery, radiation, and chemotherapy, survival rates remain low, which emphasizes the urgent need for improved therapies. Current preclinical models use untreated tumors, which do not reflect the clinical scenario where patients already receive initial treatments. This review examines the effects of current treatments on the properties of recurrent tumors and evaluates preclinical models that incorporate these standard treatments to better mimic real patient conditions. Improving these models could help to identify more effective treatments, potentially leading to better outcomes for glioblastoma patients.

**Abstract:**

Glioblastoma (GBM) presents a significant public health challenge as the deadliest and most common malignant brain tumor in adults. Despite standard-of-care treatment, which includes surgery, radiation, and chemotherapy, mortality rates are high, underscoring the critical need for advancing GBM therapy. Over the past two decades, numerous clinical trials have been performed, yet only a small fraction demonstrated a benefit, raising concerns about the predictability of current preclinical models. Traditionally, preclinical studies utilize treatment-naïve tumors, failing to model the clinical scenario where patients undergo standard-of-care treatment prior to recurrence. Recurrent GBM generally exhibits distinct molecular alterations influenced by treatment selection pressures. In this review, we discuss the impact of treatment—surgery, radiation, and chemotherapy—on GBM. We also provide a summary of treatments used in preclinical models, advocating for their integration to enhance the translation of novel strategies to improve therapeutic outcomes in GBM.

## 1. Introduction

Glioblastoma (GBM) is the most common malignant brain tumor in adults, presenting a substantial public health challenge. With an incidence of 3.2 cases per 100,000 individuals/year, approximately 12,300 new GBM cases are diagnosed annually, with peak occurrence at 66 years of age [1]. Even with the implementation of standard of care (SOC) treatment, which includes surgery, radiation therapy (RT), and chemotherapy, the 1-, 5-, and 10-year survival rates are 40.9%, 6.6%, and 4.3%, respectively [2]. These discouraging statistics underscore the urgent need for advancements in GBM therapy.

Given these challenges in GBM, reviewing the current landscape of therapeutic developments is crucial. More than 250 phase II–IV clinical trials of GBM-targeted therapies have been conducted over the past 20 years [3]. Of the 20 Phase III clinical trials from 2015 to 2019, only three met their prespecified endpoints [4]. This low number of successful clinical trials raises questions about the predictability of current preclinical models. The currently used preclinical models poorly predict treatment success in patients and overestimate the efficacy of novel anticancer agents [5]. The lack of predictive GBM models hinders the accurate assessment of a treatment’s potential efficacy and safety in human patients, leading to challenges in translating promising treatments into clinical practice.

Traditionally, novel GBM treatments are screened in preclinical models using treatment-naïve intracranial tumors [6,7]. This approach, however, does not mirror clinical practice, where patients with newly diagnosed GBM undergo resection surgery, followed by RT and chemotherapy. Unfortunately, more than half of patients present with recurring tumors within only six months after surgery [8]. A growing body of evidence indicates that recurrent tumors frequently exhibit unique mutations and subtype alterations compared to primary tumors, with many of these variations attributed to selection pressure exerted by the treatments themselves [9,10,11,12,13,14]. In the present review, we highlight the impact of each element within the SOC on the treatment timeline of GBM and the characteristics of recurrent tumors. Additionally, we provide a comprehensive overview of preclinical models integrating SOC. This approach aims to improve the identification of novel treatment strategies, bridging the gap between experimental settings and practical applications.

## 2. GBM Diagnosis and Standard of Care

Patients presenting with brain tumor symptoms such as cognitive decline, gait instability, seizures, or progressive headaches will undergo magnetic resonance imaging (MRI). Upon imaging verification of a brain mass, a biopsy/surgical resection will be obtained for pathological analysis and next-generation sequencing (NGS) to confirm GBM, determine the prognosis, and choose the most appropriate treatment regimen [15]. This process involves testing key genes such as isocitrate dehydrogenase (IDH) and O^6^-methylguanine-DNA methyltransferase (MGMT) promoter methylation, which determine tumor classification and treatment resistance. A patient’s IDH status is crucial for a GBM diagnosis, and based on the WHO 2021 classification, GBM now exclusively consists of IDH wild-type tumors [16]. Furthermore, studies have demonstrated that *IDH1* mutant tumors may have a better prognosis following surgical resection than *IDH1* wild-type tumors [17]. The MGMT status, on the other hand, is significant for treatment selection and prognosis. MGMT encodes a DNA-repair protein that removes alkyl groups. Epigenetic *MGMT* silencing through promoter methylation increases sensitivity to alkylating agents like temozolomide (TMZ), enhancing treatment efficacy and survival [18]. Thus, the MGMT status offers insights into the anticipated tumor responses to chemotherapy and guides oncologists in tailoring GBM treatment. In addition to IDH and the MGMT status, GBMs are often characterized by aberrations in the TERT promoter, chromosomes 7 and 10, EGFR, BRAF, and CDKN2A, with variable effects on the patient prognosis [16,19,20].

**Figure 1:**
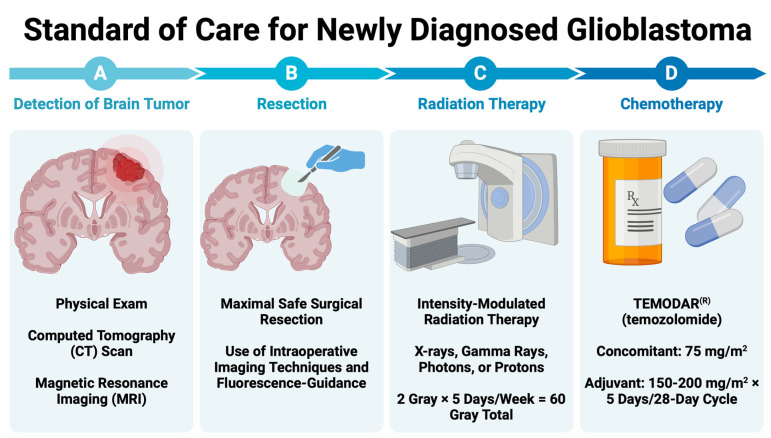
Standard of care for newly diagnosed GBM. (**A**) GBM patients often present with symptoms such as headaches, seizures, cognitive changes, or neurological deficits, which prompts a medical history review and physical examination from a healthcare professional. Tumors are typically diagnosed through a combination of imaging tests, such as MRI or CT, and biopsy. (**B**) Shortly after diagnosis, a neurosurgeon removes as much of the tumor as possible, often with the use of intraoperative imaging techniques to maximize tumor resection. (**C**) Following resection, patients undergo radiation therapy, typically receiving a total dose of 60 Gy administered in fractions of 2 Gy per session, 5 days per week. (**D**) Additionally, patients are treated with the alkylating agent temozolomide. This treatment is administered concurrently with radiation therapy at a dose of 75 mg/m^2^. Subsequently, patients undergo up to six cycles of adjuvant temozolomide, with doses ranging from 150 to 200 mg/m^2^ administered for five days in a 28-day cycle. Adapted from “Timeline 4 Steps (Layout 4 × 1)”, by BioRender.com (2024), retrieved from https://app.biorender.com/biorender-templates/figures/all (accessed on 21 February 2024).

Regardless of the sequencing results, most GBM patients receive treatment following the Stupp regimen, which has been the SOC since 2005 [21]. This regimen consists of maximal safe surgical resection, followed by RT (2 Gy × 5 days/week; 60 Gy total) and concurrent chemotherapy with TMZ (75 mg/m^2^), including up to six cycles of adjuvant TMZ (150–200 mg/m^2^ × 5 days/28-day cycle) (Figure 1). In the following sections, we describe each component of this clinical SOC.

### 2.1. Resection of Contrast-Enhancing Tissue

#### 2.1.1. Resection of Contrast-Enhancing Tissue

In maximal safe surgical resection, the neurosurgeon removes as much of the brain tumor as possible while preserving neurological function. Multiple retrospective analyses establish the relationship between the resection extent and survival [22,23,24]. McGirt et al. [22] analyzed 549 primary and 400 revision resections of malignant brain astrocytomas. In their study, the authors found that increasing the resection extent, as determined by MRI, improved median survival. Specifically, gross-total, near-total, and subtotal resection resulted in median survivals of 13, 11, and 8 months for primary tumors and 11, 9, and 5 months for recurrent tumors, respectively. For both primary and revision cases, an increased resection extent significantly improved survival (gross-total vs. near-total: *p* < 0.05; near-total vs. subtotal: *p* < 0.05). Lacroix et al. [23] demonstrated that resecting more than 98% of contrast-enhancing tumor volume significantly improves survival compared to resection of less than 98% (13 vs. 8.8 months; *p* < 0.0001). Furthermore, recent data from Sanai et al. [24] revealed survival benefits even at 78% resection, with stepwise improvements upon resection of 90%, 95%, 98%, and 100% of the contrast-enhancing tumor (100%: 16 months vs. overall: 12.2 months), highlighting the importance of the resection extent in the SOC for GBM patients.

#### 2.1.2. Resection of Non-Contrast-Enhancing Tissue

Researchers also examined the survival of patients after additional non-contrast-enhancing tumor resection, a procedure referred to as supramarginal resection. Given the distinct characteristics of *IDH1* mutant and *IDH1* wild-type tumors, Beiko and colleagues [17] examined the impact of *IDH1* mutation on the surgical resection and survival outcomes of patients with glioma. The researchers found that compared to *IDH1* wild-type tumors, *IDH1* mutant tumors are more conducive to surgical resection, with complete resections achieved in 93% of *IDH1* mutant tumors vs. only 67% of *IDH1* wild-type tumors. Furthermore, supramarginal resection conferred a survival benefit in only patients with *IDH1* mutant tumors [17]. Molinaro et al. [25] also examined the survival effect of supramarginal resection within *IDH* wild-type and *IDH* mutant GBM subgroups. Despite a historically lower median survival of patients with IDH wild-type tumors compared to IDH-mutant tumors (1.2 vs. 3.6 years) [26,27], supramarginal resection in patients < 65 years with IDH wild-type GBM led to a median overall survival comparable to IDH mutant tumors. Additionally, supramarginal resection resulted in significantly longer survival than resection of only the contrast-enhancing tumor (37.3 vs. 16.5 months, HR 0.36, 95% CI 0.25–0.51, *p* < 0.001), and this improved survival was present in all subtypes. In contrast to Beiko et al., the percentage of complete resections achieved did not differ based on *IDH1* status (89.9% vs. 89.5%, *p* = 0.90). Consequently, for patients under the age of 65 years, the authors recommend maximal resection of both contrast-enhancing and, when safely feasible, non-contrast-enhancing tumor tissue.

#### 2.1.3. Resection Using Fluorescent Imaging Agents

Fluorescent contrast agents are becoming a standard feature in neurosurgical oncology, aiding surgeons in intraoperative differentiation between tumor and healthy tissue. The most common optical imaging agent is 5-aminolevulinic acid (Gleolan^®^, 5-ALA), which is a non-fluorescent prodrug in the porphyrin synthesis pathway that is converted into the fluorescent heme precursor, protoporphyrin IX [28]. Protoporphyrin IX preferentially accumulates in tumor tissue due to variations in enzyme and transporter expression levels and regions of a leaky blood–tumor barrier, thus allowing visualization with a fluorescence microscope [29,30,31].

In a phase III randomized controlled trial with 322 glioma patients, neurosurgeons conducting 5-ALA-guided resection removed all contrast-enhancing tissue in 65% of cases, a higher rate compared to 36% in cases using white light (difference between groups = 29%, 95% CI 17–40, *p* = 0.0001) [28]. In this trial, 6-month progression-free survival was significantly improved in the 5-ALA group compared to the control group (41.0% vs. 21.1%, difference between groups = 19.9%, 95% CI 9.1–30.7, *p* = 0.0003). Notably, through patient selection, randomization, and balancing, coupled with the impact of 5-ALA, the authors provided definitive evidence regarding the importance of complete resection in a retrospective post hoc analysis of the 5-ALA study [32]. In a multivariate analysis by Stupp et al., the residual tumor, age, and Karnofsky Performance Scale were revealed to be significant prognostic factors. However, upon further patient stratification, only complete resection—rather than age or tumor location—remained significant, providing level IIb evidence of the significant survival benefit associated with complete resection of enhancing tumor tissue. In terms of safety, groups did not differ significantly in the frequency of adverse effects, such as hemiparesis (5-ALA: 3% vs. white light: 2%, *p* = 0.7), aphasia (2% vs. 0%, *p* = 0.1), convulsions (2% vs. 1%, *p* = 0.6), and epidural hematoma (1% vs. 1%, *p* = 1.0). Laboratory measurements were increased in patients who had received 5-ALA compared to white light resection only after 24 h (gamma glutamyl transpeptidase: 0.93× vs. 0.72× upper limit of normal (*p* = 0.05), alanine transaminase: 1.05× vs. 0.84× upper limit of normal (*p* = 0.003), and aspartate aminotransferase: 0.72× vs. 0.53× upper limit of normal (*p* < 0.0001)). Stroke scores for patients who received 5-ALA worsened or did not improve to a greater extent compared to those who received white light resection 48 h following surgery. Deterioration was noted in 24% of the 5-ALA group compared to 15% of the white light group, while improvement was seen in 24% of the 5-ALA group compared to 31% of the white light group (*p* = 0.0462). However, the groups did not differ significantly at 7 days or 6 weeks following surgery.

For patients receiving 5-ALA, it has been suggested that improved survival may result from better visualizing fluorescent tumor tissue beyond the boundaries identified using MRI, revealing areas with invasive GBM cells. In a retrospective observational study of patients who underwent 5-ALA-guided tumor resection, the mean overall resection volume was significantly greater than the volume of contrast-enhancing tumor (84 cm^3^ vs. 39 cm^3^, 95% CI −30.3 to 10.4, *p* = 0.32), suggesting that 5-ALA-positive tissue extends beyond contrast-enhancing tissue [33]. However, this study only included 13 patients. Furthermore, in this study, it is unclear whether the survival benefits of 5-ALA came from its ability to identify and remove more invasive tumor cells that are not visible on MRI (supramarginal resection) or if it primarily helped surgeons achieve a complete removal of the tumor as defined by standard imaging criteria.

Fluorescein sodium is another fluorescent imaging agent used in glioma surgery. A recent systematic review demonstrated that the odds of achieving gross total resection, defined as >95% of contrast-enhancing tissue, are significantly higher with fluorescein sodium (4.0-fold) and 5-ALA (3.4-fold) compared to white light resection (*p* < 0.01) [34]. However, no significant difference in gross total resection rates was observed between fluorescein sodium and 5-ALA. Given the potential of targeted fluorescent agents for GBM surgery, more such agents are currently under development [35].

### 2.2. Radiation Therapy (RT)

About 4 to 6 weeks following surgery, patients receive RT [21,36]. The addition of RT to surgery and chemotherapy extends survival from 3–4 months to 7–12 months, surpassing the outcomes of surgery or chemotherapy alone [21,37,38]. A total radiation dose of 60 gray, which is delivered to the tumor, and a 1–2.5 cm margin of non-contrast-enhancing tissue, is given over 5 days/week for 6 weeks, with individual doses ranging from 1.8 to 2.0 gray [39].

#### RT Techniques

Common RT delivery methods include 3D conformal RT and intensity-modulated RT. The former utilizes imaging (e.g., CT or MRI) to create a 3D image of the patient’s tumor, enabling precise tumor targeting while minimizing radiation to surrounding brain tissue [40]. Intensity-modulated RT employs advanced planning and delivery software to shape photon and proton beams and adjust dose intensity across treatment fields, which is beneficial when tumors are near radiation-sensitive structures like the eyes, optic nerves, or brainstem [41]. In a retrospective study comparing intensity-modulated RT and 3D conformal RT, no significant difference in survival was observed [42]. However, intensity-modulated RT demonstrated lower incidences of disorders affecting concentration (28.4 vs. 11.5%, *p* = 0.007) and consciousness (20.4 vs. 4.1%, *p* = 0.004).

Less commonly studied RT techniques in GBM include interstitial brachytherapy and proton beam RT. Interstitial brachytherapy involves placing radioisotope seeds (often I-125, 192-Ir, or Cs-131) in the tumor or resection cavity, releasing low-dose-rate radiation within a few millimeters of the seed over time. Given the highly invasive nature of GBM, a comprehensive review of 1571 GBM patients found little to no survival benefit with brachytherapy compared to traditional RT [43]. Proton beam RT is a particle RT. The advantage of proton therapy over traditional RT is that the protons have a finite path length and concentrate most of their dose on the tumor [44], allowing greater precision in tumor treatment and decreasing exit radiation exposure to normal structures. In a recent phase II randomized controlled trial with 67 patients comparing proton RT and intensity-modulated RT, no significant differences in cognitive failure, overall survival, or intracranial progression-free survival were noted [45]. However, proton RT significantly reduced radiation exposure in all adjacent structures. Despite this benefit, recent studies have indicated a higher incidence of radiation necrosis following proton beam therapy due to the variable biological effectiveness of protons, which is not adequately accounted for in current clinical practice [46,47,48]. Additional studies are warranted to further understand proton-therapy-associated radiation necrosis and develop treatment strategies to prevent this side effect. A randomized phase II dose-escalation trial using photon intensity-modulated RT or proton RT for treating patients with newly diagnosed GBM is ongoing [49].

### 2.3. Chemotherapy

Alongside RT, GBM patients usually receive daily TMZ chemotherapy (75 mg/m^2^) for six weeks. This is followed by a four-week break and up to six cycles of TMZ (150–200 mg/m^2^ for 5 days every 28 days) alone [21]. Evaluation of MGMT promoter methylation is part of the standard of care for GBM patients, as it determines their sensitivity to TMZ. MGMT promoter methylation results in lower MGMT DNA repair enzyme expression, making the tumor sensitive to alkylating agents like TMZ, and is predictive of improved survival [50]. TMZ efficacy was initially demonstrated in a trial by Dr. Roger Stupp and colleagues [21]. In this trial, 573 patients were randomized to receive either RT alone (2 Gy × 5 days/week for six weeks; 60 Gy total) or a combination of RT and concurrent daily TMZ (six weeks of 75 mg/m^2^) followed by up to six monthly cycles of TMZ (150–200 mg/m^2^ × 5 days per 28-day cycle). After a median follow-up time of 28 months, RT with concurrent TMZ increased median survival (14.6 vs. 12.1 months; HR 0.63, 95% CI 0.53–0.75) and the two-year survival rate (26.5 vs. 10.4%) compared to RT alone. This protocol (“Stupp protocol”) was widely adapted and is currently the SOC for newly diagnosed GBM patients.

The Stupp trial also investigated the impact of the MGMT methylation status on TMZ sensitivity. In a group of 206 patients, MGMT methylation significantly increased survival for patients receiving TMZ and RT compared to RT alone (21.7 vs. 15.3 months, HR 0.45, 95% CI 0.32–0.61) [18]. Among patients without a methylated MGMT promoter, TMZ/RT treatment insignificantly increased median survival (12.7 vs. 11.8, HR 0.69, 95% CI 0.47–1.02). While the MGMT status did not determine the treatment in the Stupp trial, patients with unmethylated MGMT are often directed to clinical trial therapy, which incorporates novel therapies instead of TMZ.

Alternative treatments, such as intratumoral oncolytic viral therapies [51], medicated wafers [52], and immunotherapies [53], have also been explored. The survival benefits observed with these strategies have varied, but encouraging results suggest that further research is warranted to fully understand their potential and optimize their use.

## 3. The Effect of the Standard of Care on GBM

While SOC therapy increases patient survival, it also influences the disease trajectory, leading to distinct changes between primary and recurrent tumors [10,12,13,14,54,55,56]. Johnson et al. [10] sequenced 23 glioma pairs and found that over 50% of mutations in primary tumors were undetectable in recurrent tumors. This finding suggests early tumor seeding following treatment, allowing time for the development of genetically distinct tumor populations. Additionally, 60% (6/10) of recurrent tumors exhibited a TMZ-induced hypermutated phenotype with retinoblastoma and Akt-mTOR gene mutations. Kim et al. [57] found that Stupp regimen therapy induces hypermutations in recurrent tumors, demonstrating both linear and divergent evolution from the primary tumor. The study highlights that p53 pathway mutations increase the frequency of subclonal mutations, which are unique genetic changes within specific subsets of tumor cells. Wang et al. [55] similarly explored GBM tumor evolution, revealing a highly branched evolution pattern and enriched mutational switching. Mutational switching occurs when a different mutated version of the same gene replaces one mutated version of a gene. Interestingly, mutational switching increases about 200-fold in genes commonly mutated in GBM, including *EGFR*, *TP53*, and *PDGFRA*. Recurrent GBM-specific mutations included *MSH6* and *LTBP4*, encoding a mismatch repair protein and TGF-β regulator.

In contrast to earlier research, Muscat et al. [56] also included tumor samples from control patients who underwent resection surgery but no other therapies. Tumors that recurred after surgery alone exhibited a neutral evolutionary pattern. In contrast, recurrent tumors following surgery combined with radiation therapy or chemotherapy showed non-neutral evolution, characterized by a non-linear relationship between subclonal variants and their frequency. Notably, the proportion of recurrent tumors with a TMZ-induced hypermutated phenotype reported by Muscat et al. (4%) [56] was lower than those reported by Wang et al. (17%) [55], Kim et al. (24%) [57], and Johnson et al. (60%) [10].

Another important consideration is the concept of the tumor mutation burden, which quantifies the overall number of mutations within the tumor DNA. Zhang et al. [12] performed hybrid capture-based next-generation sequencing on 64 primary and 17 recurrent tumor samples, revealing more copy number variations, co-occurrence of IDH1 and TERT mutations, inactivated cell cycle signaling, and a higher tumor mutation burden in recurrent tumors. Interestingly, these researchers observed a direct relationship between the tumor mutation burden and patient prognosis, which could be explained by increased immunogenicity of tumors with higher mutational loads.

Another intriguing aspect of cancer progression is phenotype switching, which involves dynamic changes in a tumor’s physical and biochemical characteristics. Wang et al. [13] investigated the impact of therapy on GBM plasticity, revealing a shift from a proneural phenotype in primary tumors to a mesenchymal phenotype in recurrent tumors. Furthermore, these researchers discovered pro-tumor paracrine and autocrine signals between GBM cells, neuroglia, and immune cells that persisted or were upregulated in recurrent GBM. Hoogstrate et al. [14] also studied the effect of the tumor microenvironment on tumor recurrence and showed a decrease in tumor purity and endothelial marker genes paired with an increase in neuro, oligodendrocyte, and tumor-associated macrophages. These trends suggest that microenvironment reorganization, in addition to molecular evolution, primarily influences GBM progression throughout treatment.

**Figure 2:**
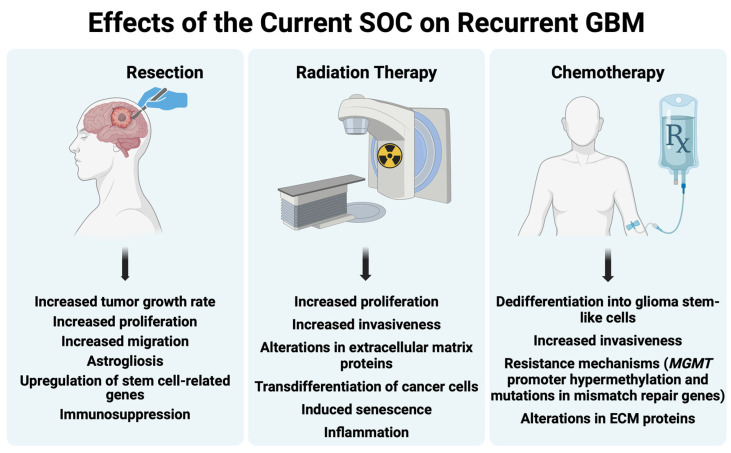
Effects of the current standard of care on recurrent glioblastoma. Primary effects of resection, radiation therapy, and chemotherapy on recurrent GBM. Created with BioRender.com.

In summary, the findings from these sequencing studies demonstrate the presence of unique mutations in recurrent tumors compared to untreated tumors. Moreover, the current SOC for GBM may contribute to the differentiation between primary and recurrent GBM. In the following three sections, we describe the specific effects of surgery, RT, and chemotherapy on the characteristics of recurrent tumors and the tumor microenvironment (see Figure 2 for a summary).

### 3.1. Surgery—Effects on Recurrent GBM

In this section, we assess the impact of surgery (biopsy and resection) on GBM, considering its effects on growth, migration, microenvironment, gene expression, and immune response. Additionally, we discuss the influence of anesthesia on patient survival.

#### 3.1.1. Biopsy

To assess the effect biopsy has on GBM growth, Weil et al. [58] used longitudinal in vivo two-photon confocal microscopy to follow tumor growth and the fate of single patient-derived GBM stem-like cells in nude mice. These researchers generated a surgical lesion model by removing a cylindrical volume (~300 µm diameter) from the established tumor using a 26-gauge needle. Three days following surgery, GBM stem-like cells extended ultra-long (>50 µm) tumor microtubes towards the lesion, with an increasing percentage of tumor microtubes directed towards the lesion at 7 days post-surgery. Targeting growth-associated protein-43, a protein critical for microtube formation, inhibited microtube formation and suppressed post-surgical tumor growth. Furthermore, microtube-connected GBM stem-like cells demonstrated increased resistance to TMZ treatment, with a higher survival rate observed in cells extending more than four microtubes compared to those with fewer projections at 62 days after TMZ treatment. However, surgery was not conducted in the TMZ treatment studies. Using a similar technique, Alieva et al. [59] extracted part of GL261 tumors from mice using a 25-gauge needle. Cancer cell migration and proliferation were significantly increased at 24 h after biopsy compared to unbiopsied tumors. These alterations were driven by chemokine ligand 2-dependent macrophage recruitment. Further studies are needed to determine the duration of these biopsy-dependent effects, with and without additional standard-of-care treatments.

Data from patient samples also demonstrate biopsy effects on tumor growth dynamics [59]. In a retrospective analysis of multifocal GBM patients with a biopsy of only one tumor site, the volume of the biopsied tumor increased more than that of non-biopsied tumors. Dexamethasone pretreatment decreased biopsy-induced tumor progression, indicating that inflammation might play a role in this process [59].

#### 3.1.2. Resection

In clinical practice, biopsies are usually taken during resection rather than through needle biopsy. Accordingly, resection models have been developed to investigate the impact resection has on GBM tumors. Okolie et al. [60] removed >90% of TRP-mCherry-FLuc tumors from mice using image-guided microsurgery. Resection significantly increased the tumor growth rate, with doubling times of 3.3 days (pre-resection) and 1.9 days (post-resection; *p* = 0.0003). Further, surgery altered the temporal and spatial characteristics of reactive astrocytes in the peritumoral microenvironment. Increased levels of the chemokine CXCL5 suggest that astrocytic injury signals through CXCL5 to enhance tumor invasion and proliferation after GBM resection; however, these findings need further research. Other groups have demonstrated that reactive astrogliosis following surgery increases paracrine factors such as GF-α, CXCL12, S1P, GDNF, MMP-2, and MMP-9 that could also contribute to altered GBM growth and migration [61,62,63]. Knudsen et al. [64] extended these findings in mice using bulk and single-cell RNA sequencing to identify distinct gene expression and pathway signatures in recurrent tumors within a rat GBM resection model and early recurrent patient tumors. Compared to primary tumors, recurrent tumors exhibited increased proliferation, higher degrees of angiogenesis, greater microglia/macrophage infiltration, and elevated levels of stem-cell-related proteins (SOX2, OLIG2, POU3F2, and NOTCH1). Pleiotrophin mRNA and protein levels were also elevated in recurrent rat and patient tumors, associated with poor overall survival.

Additional research focused on the immune response following neurosurgical operations. Given that non-CNS cancer surgeries induce an immunosuppressive tumor microenvironment [65], Sablotzki et al. [66] measured plasma cytokine levels and lymphocyte subsets from patients with either GBM or intracerebral aneurysms. Neurosurgery significantly increased plasma IL-10 and TGF-β in GBM patients but not in aneurysm patients. To maintain consistency between groups, all patients received 7.5 mg midazolam 45 min before surgery, weight-related doses of anesthesia, and antimicrobial prophylaxis with 2 g cefamandole. Nearly all patients received perioperative corticoids (hydrocortisone 3 × 4 mg/day). Furthermore, Otvos et al. [67] demonstrated that GL261 and CT-2A glioma resection reduced circulating T cells and increased CD8+ T cells in the bone marrow compared to mock resection, which involved corticectomy, opening of the dura, and removal of white matter above the tumor. These data reiterate that surgery suppresses the immune system, including brain tumor resections.

#### 3.1.3. Anesthesia

While surgery-induced trauma increases GBM growth characteristics and alters the tumor microenvironment, anesthesia affects GBM recurrence and metastasis. Specifically, in GBM resection patients, propofol increased survival (HR 0.51, 95% CI 0.30–0.85) and decreased postoperative tumor recurrence (HR 0.60, 95% CI 0.37–0.98) compared to desflurane [68]. In a retrospective cohort study of 154 patients receiving propofol and 140 patients receiving sevoflurane, median progression-free and overall survival were not significantly different. However, sevoflurane increased the death risk in patients with a Karnofsky performance status <80 compared to propofol (HR 1.66, 95% CI 1.08–2.57) [69]. Survival differences might be linked to the attenuation of the surgery-induced adverse immune response with propofol anesthesia [70,71]. In contrast, other researchers found no survival or recurrence time differences between volatile and IV (propofol) anesthetics in GBM surgery patients, suggesting that more studies are needed to assess whether anesthesia affects GBM recurrence and survival [72,73].

Volatile anesthetics promote tumorigenesis through various mechanisms in different cancers (extensively reviewed in [74]). For example, sevoflurane increases levels of tumorigenic cytokines, MMPs, and HIF-1α while decreasing levels of polymorphonuclear cells, NK cell activation, IL-1β, and TNF-α [75,76,77]. Similarly, isoflurane activates HIF-1α and enhances glycolysis, angiogenesis, and the expression of VEGF, IGF-1, IGF-1R, angiopoietin-1, MMP-2, and MMP-9 [78,79]. In contrast, propofol blocks HIF-1α induction, suggesting a difference in mechanisms between inhaled and intravenous anesthetics [79]. In summary, surgery for tumor resection significantly alters GBM growth, migration, microenvironment, gene expression, and immune reactivity. While some observational data indicate anesthesia may influence survival and recurrence rate in GBM, further investigations are needed to better understand the observed survival outcomes following resection under anesthesia.

### 3.2. Radiation Therapy—Effects on Recurrent GBM

The effect RT has on the GBM microenvironment is well-documented [11]. This section provides a concise overview of the genotypic and phenotypic alternations RT induces in GBM tumors and their surrounding microenvironment.

#### 3.2.1. Effects on GBM Tumor

##### Genotypic and Phenotypic Alterations

Similar to surgery, RT influences GBM tumor properties and growth characteristics. Muthukrishnan et al. [80] conducted single-cell and whole transcriptomic analysis and demonstrated that GBM cells undergo trans-differentiation, acquiring vascular- and mesenchymal-like phenotypes following RT. This phenotypic shift resulted from increased vascular gene accessibility and was inhibited with p300 histone deacetylase inhibitors. Mahabir et al. [81] also observed a transition from a glial to mesenchymal phenotype using IHC and qRT-PCR in primary and recurrent glioma samples, cell lines, and primary glioma cells. Recurrent tumor samples exhibited elevated levels of proteins involved in mesenchymal functions, including collagen, MMPs, and YKL-40, accompanied by increased Snail-dependent migration, invasion, focal adhesion number, and MMP-2 levels. In NSC11 and NSC20 tumor-bearing mice, RT (3 × 5 Gy) resulted in less invasive tumors with distinct mutation patterns, including defective DNA repair and BRCA1/2 mutations not found in controls [82]. Additionally, sub-cytotoxic ionizing radiation enhanced stem-like properties and tumorigenicity in patient GBM cells [83] and increased invasiveness in the 9L rat glioma model, as indicated by the tumor cell satellite number [84]. This change in invasiveness contradicts the study by McAbee et al. [82], suggesting that RT effects might be model-dependent, especially regarding stem-like cells vs. differentiated cells.

##### Metabolic Alterations

Implanting human xenografts (GBM143) in irradiated mice yielded more proliferative and invasive tumors than in non-irradiated mice, suggesting that RT triggers alterations in the microenvironment that favor tumor growth [85]. The tumors from irradiated mice had higher ATP and GTP levels and reduced levels of antioxidants (ascorbate and glutathione) compared to tumors from mice that received no radiation. Additionally, researchers have shown alterations in glucose metabolism. Shen et al. [86] showed that RT shifts U87 cells from oxidative phosphorylation to glycolysis, and the increased reliance on glycolysis enhanced the GBM cells’ tumorigenic potential. Bailleul et al. [87] recently published on the role glucose metabolism plays in radioresistance, showing increased GBM cell glucose utilization with GLUT3 transporter translocation post-RT. Glucose is then routed through the pentose phosphate pathway, supporting NADPH and ribose-5-phosphate generation for antioxidant defense and nucleotide synthesis. De Martino et al. [88] also found that RT influences fatty acids. Specifically, RT increased fatty acid accumulation, leading to lipid droplet formation and the synthesis of bioactive lipid compounds, specifically prostaglandin E2, which researchers have shown to be associated with cancer stemness [89]. This increased lipid metabolism was confirmed in recurrent tumors analyzed from the GLASS consortium dataset.

To determine the upstream mediators of these changes, Jeon et al. [90] demonstrated that RT induces senescence of GBM cells, which are characterized by high tissue factor (CD142) expression. The tissue factor, in turn, promotes a transition to a mesenchymal-like cell state, chemokine secretion, tumor-associated macrophage activation, and extracellular matrix remodeling, which is discussed in more detail in the next section.

#### 3.2.2. Effects on Tumor Microenvironment

##### Extracellular Matrix

Following RT, several extracellular matrix proteins, including collagen, tenascin C, brevican, and vitronectin, are upregulated, which contributes to increased tumor proliferation, migration, and invasion. These effects are consistent with the extracellular matrix playing a role in angiogenesis, structural support, cell adhesion, and therapeutic resistance [reviewed by [11]]. Additionally, RT induces stromal cell senescence, promoting GBM growth through receptor tyrosine kinase activation [91]. Increased expression levels of other extracellular matrix proteins like hyaluronic acid post-RT yield a pro-invasive microenvironment and signal that GBM cells shift toward a mesenchymal phenotype [92].

##### Vasculature

Since RT affects proteins involved in angiogenesis, it also affects the vasculature. In an orthotopic glioma stem cell tumor model, Seo et al. [93] found that a single dose of 10 Gy cranial irradiation resulted in abnormal vasculature in the irradiated tumors vs. non-irradiated tumors. This abnormal vasculature was characterized by a lower microvessel density, vascular dilation, and a significant decrease in the apparent diffusion coefficient, which is a measure of water diffusion in tissue. Moreover, in recurrent GBM patients, prior RT increased intracavitary fluid VEGF levels compared to those without prior RT [94]. These RT-dependent increases in VEGF were further linked to elevated cell migration and invasion in vitro [95].

A systematic review and meta-analysis of 69 studies found that 35% (7/20) of the clinical studies reported blood–brain barrier disruption following RT, with acute effects occurring 3 to 4 weeks following RT [96,97]. Preclinical studies showed a higher frequency, with 78% (38/49) demonstrating disruption after RT. Mechanisms by which RT disrupts the blood–brain barrier include the downregulation of claudin-5 and increases in the cytokine TNF-α and the chemokine CXCL1 [98,99]. In wild-type mice, various inflammatory mediators correlated with blood–brain barrier disruption. Specifically, TNF-α levels rose immediately following RT and returned to baseline within 6 h, while CXCL1 levels significantly increased starting at 6 h post-RT and continued through to 12 h post-RT [99].

##### Immune System

Lastly, RT triggers immune and inflammatory reactions [100]. A single RT dose of 15 gray to the whole rat brain increased glial fibrillary acidic protein levels, indicating astrocytic gliosis. Furthermore, RT increased mRNA levels of COX-2, IL-1β, IL-6, IL-18, TNFα, and IP-10 in mouse microglial cells [101] and IL1A, CXCL1, IL-6, and IL-8 in U87-bearing mice [102]. In humans, RT elevated IL-6, IL-8, monocyte chemoattractant protein-1, and macrophage inflammatory protein-1 alpha levels about 2- to 3-fold in comparison to baseline levels [103].

These data highlight the transformative impact RT has on GBM tumors and their microenvironments, ranging from metabolic adaptations and extracellular matrix modifications to vascular irregularities and immune responses. The interplay of these changes underscores the dynamic nature of the post-RT GBM biology and emphasizes the critical need to incorporate the nuanced effects RT has in preclinical studies and models, ensuring that therapeutic strategies are developed within the evolving tumor microenvironment for accurate and effective translational outcomes.

### 3.3. Chemotherapy—Effects on GBM

Since TMZ is the most commonly used FDA-approved cytotoxic drug in the treatment of GBM, we will focus on the effects TMZ has on recurrent GBM tumors. We briefly discuss TMZ-induced alterations in cellular differentiation, resistance mechanisms, and the tumor microenvironment.

#### 3.3.1. Cellular Differentiation

Data from one of the earliest reports on TMZ-induced changes in GBM show dedifferentiation of non-stem glioma cells into glioma stem-like cells. Exposure of patient-derived glioma cells to TMZ significantly increased glioma stem-like cells in vitro and in vivo. TMZ-treated cells resulted in tumors with greater tumor take and increased invasiveness [104]. Later studies suggest a role for TMZ-induced hypoxia-inducible factors 1α and 2α in this phenotypic switch [105].

#### 3.3.2. Drug Resistance

Phenotypic changes in cancer cells can contribute to cellular drug resistance, triggering research studying the TMZ effect on resistance mechanisms of recurrent GBM tumors. In this regard, in TMZ-resistant cells with over 50% *MGMT* promoter hypermethylation, TMZ decreases hypermethylation and enhances migratory capacity [106]. In TMZ-sensitive cells with no *MGMT* promoter methylation, the proliferation rate increases. Mismatch repair proteins, particularly MSH6, show inactivating mutations in recurrent GBM samples, suggesting a role in resistance [107]. Reconstituting MSH6 increases TMZ sensitivity in MSH6-null primary GBM cells, suggesting that mismatch repair defects are an important mechanism leading to resistance in GBM with inactive MGMT [108]. Additionally, TMZ treatment increases the expression of programmed death-ligand 1 (PD-L1) on the GBM cell membrane, contributing to immune escape. Co-treatment with TMZ and actinomycin or STAT3 inhibitor VI attenuates PD-L1 expression, indicating transcription- and STAT3-dependent upregulation [109].

#### 3.3.3. Tumor Microenvironment

TMZ also influences the extracellular matrix and, thus, the tumor microenvironment. In ex vivo brain organotypic slices and in vivo xenograft GBM tumors, TMZ altered the brain extracellular matrix proteoglycan composition, which resulted in increased proliferation and invasion of GBM cells. Of the altered proteoglycans, chondroitin sulfate led to the greatest changes in phenotype [110].

In summary, TMZ induces dedifferentiation, influences resistance mechanisms involving altered methylation patterns and mismatch repair defects, and alters the tumor microenvironment.

## 4. Preclinical Models Recapitulating GBM Standard of Care

Since surgery, RT, and chemotherapy are the gold standard for GBM patients, and each component affects residual disease, incorporating these treatments into preclinical studies could increase the translational value of novel research approaches. In the following section, we summarize the use of resection, RT, and chemotherapy in animal models and highlight combinations of the three modalities.

Resection is the first component in the SOC for GBM treatment, and previous data underscore its impact on GBM growth, gene expression, the microenvironment, and immune cell profiles. These effects emphasize the need to replicate resection in animal models. We will review preclinical surgery techniques, including white light resection, biopsy, and fluorescence-guided resection, focusing on their application in preclinical experiments and translation.

### 4.1. Resection

#### 4.1.1. White Light Resection

In white light resection, surgeons use regular white light (vs. fluorescent, infrared, or laser light) to remove brain tumors, relying on visual differences between tumor and normal tissue. Tang et al. [111] used a combination of blunt and sharp dissection with the aid of an operating microscope to resect approximately 85% of the tumor mass from GL261-bearing mice 16 days post-implantation, resulting in a 5-day increase in median survival (34 vs. 39 days, *p* = 0.0896). Despite using an operating microscope for tumor visualization, challenges in white light resection include difficulty distinguishing between tumor and healthy tissue, especially in small-scale applications with animal models [112]. This publication is a unique instance where the authors exclusively use white light for resection without concurrent therapies, underscoring the need for more research.

#### 4.1.2. Punch Biopsy

In comparison to white light resection, some groups use a quicker approach of applying a punch biopsy tool for tumor removal. On day 13 post-implantation, Bianco et al. [113] resected U87 tumors using a 2 mm diameter punch biopsy tool. The biopsied tissue, obtained by twisting the punch biopsy tool to a depth of 3 mm, was aspirated using a Pasteur pipette attached to a vacuum pump. Tumor resection increased median survival from 24 days to 36 days, a significant increase compared to mice without resection (*p* = 0.0021).

Other researchers used similar techniques to create a resection cavity suitable for local drug delivery systems, as discussed in the Resection + TMZ section below [114,115]. The advantages of using a punch biopsy tool over white light resection include shorter procedure times and consistent volumes of resected tissue. However, the punch biopsy approach may be less aggressive depending on tumor size, potentially leaving residual tumor tissue.

#### 4.1.3. Fluorescence-Guided Resection: Fluorescent-Labeled Cells

To maximize tumor removal while sparing healthy brain tissue, researchers use fluorescent tags such as GFP and mCherry for tumor visualization. Kauer et al. [116] removed about 60% and 80% of U87-mCherry tumors in mice injected with 7.5 × 10^4^ cells (small tumors) and 1.5 × 10^5^ cells (large tumors), respectively, as assessed by bioluminescence imaging. Resection on day 14 post-implantation increased survival from 30.5 to 40 days for 7.5 × 10^4^ injected cells and from 25.5 to 33 days on day 21 post-implantation for 1.5 × 10^5^ injected cells. Sheets et al. [117] used a similar technique with a fluorescence dissecting stereomicroscope, gently aspirating tumors 7 days after implanting 10^5^ U87-mCherry-FLuc cells into the brains of nude mice. This methods paper did not report the extent of resection or survival outcomes. Rogers et al. [118] used a similar process for serial biopsy to assess the treatment response. For both primary and secondary biopsies, U87-GFP-Luc2 tumors were detected using a digital camera microscope, followed by tissue biopsy using forceps and microdissection scissors. The second biopsy occurred after the mice fully recovered from the initial surgery and 2 h after the EGFR inhibitor dacomitinib was administered. This approach revealed reduced EGFR phosphorylation in post-treatment compared to pre-treatment histopathology samples.

Similar to a punch biopsy, fluorescence-guided resection has also been combined with other treatments that line the resection cavity, including chemotherapy and encapsulated stem cells, which are discussed in the Resection and Chemotherapy section. Although fluorescent tags help maximize the extent of resection for research purposes, this does not mirror clinical practice, as GBM tumor cells do not naturally fluoresce. Therefore, fluorescent imaging agents are used, as discussed in the following section.

#### 4.1.4. Fluorescence-Guided Resection: Fluorescent Imaging Agents

Fluorescent imaging agents, particularly 5-ALA, have been used for tumor resections in preclinical tumor models as well as in GBM patients. Fluorescence of tumor tissue after 5-ALA administration has been demonstrated in rabbit (VX2), mouse (GL261 and U87), and rat (C6) glioma models [119,120,121,122]. Previous studies have used 5-ALA ranging from 100 mg/kg to 200 mg/kg without observed toxicity. Higher doses, up to 1000 mg/kg, have also been used without apparent adverse effects in the photodynamic therapy of oral lesions in mice [123].

In rabbits harboring intracranial VX2 tumors, 5-ALA-guided surgery enhanced tumor visualization, resulting in a 1.4-fold increase in resection extent and a 16-fold decrease in residual tumor volume compared to white light resection, as measured by histopathology [124]. Similarly, in mice with U87-GFP-FLuc tumors, researchers achieved over 90% tumor removal using 5-ALA guidance [121]. Colocalization between 5-ALA and GFP fluorescence was observed, indicating accurate targeting of GFP-tagged tumor cells.

Fluorophores less commonly used in resection surgery include fluorescein sodium [125] and indocyanine green (ICG) [126,127,128]. While fluorescein sodium and ICG are nonspecific imaging agents that primarily rely on a disrupted blood–tumor barrier to accumulate within the tumor, more targeted imaging agents are studied in preclinical models. These agents include metabolic agents, antibody-targeted, peptide- or phospholipid-targeted, or enzymatic-activity-based probes. Some of the most prevalent agents include IRDye 800CW-RGD (binds integrin receptor), anti-TRP-1-Alexa fluor 488 or 750, anti-EGFR antibodies, and Angiopep-2-Cy5.5 (binds LRP). Their targets are listed in Table 1 [35,129].

Exploring various approaches to GBM surgery in preclinical models shows that each technique brings unique advantages and challenges. With white light resection requiring fewer resources than other techniques, it is difficult to distinguish tumor from healthy tissue, particularly in small animal models. Punch biopsy is a quick procedure that is easily reproducible but leaves behind residual tumor tissue, impacting outcomes. Fluorescence-guided resection with fluorescent-labeled cells, while effective for research purposes, does not mirror clinical practice due to the absence of natural fluorescence in GBM tumor cells. However, fluorescent imaging agents, including 5-ALA, have shown promise in enhancing tumor visualization and achieving precise resection. The combination of preclinical surgical approaches with other components of the GBM SOC is discussed below.

### 4.2. Radiation Therapy

Initially, RT in preclinical models was limited to whole-brain radiation due to a lack of radiation precision of early technology [130]. Whole-brain RT in mice disrupts the blood–brain barrier [96], alters the microglial landscape [131], impairs cognition, and damages astrocytic calcium signaling [132]. With technological advancements, particularly the introduction of small animal irradiation systems, radiation precision in preclinical animal models has improved. Rutherford et al. [133] treated G7 GBM xenograft mice with four different modalities: a single beam, parallel opposed pair, single plane arcs, and couch rotation arcs. Dose delivery accuracy was measured using an imaging phantom. In all treatment groups, radiation doses were achieved at the tumor while avoiding other organs. Deng et al. [134] added 3D bioluminescence tomography to determine the tumor center of mass, to target the radiation. Survival was not measured in these studies, but γ-H2AX staining to detect double-strand breaks was seen in regions determined to be tumor by MRI, suggesting appropriate radiation targeting and delivery.

Using these RT techniques that are more precise than earlier techniques, Stackhouse et al. [135] generated eight GBM patient-derived xenograft models with acquired RT resistance from matched treatment-naïve, RT-sensitive models. Sensitivity studies involved irradiating mice with 6 × 2 Gy over two weeks. Radiosensitive tumors then underwent serial selection for 6–8 additional RT rounds. Transcriptomic and kinomic profiling of RT-resistant models revealed enrichment of DNA damage repair proteins, correlating with specific long noncoding RNAs and targetable kinases. This set of novel paired models provides insights into RT resistance and holds the promise to be a valuable tool for future research. Zhou et al. [136] also used multiple models to study RT resistance. Specifically, their data from targeted metabolomic studies show high purine levels related to RT resistance in 23 GBM cell lines. Inhibiting GTP synthesis with mycophenolate mofetil enhanced RT effects in GBM38 mice, resulting in significantly longer median survival compared to control mice, mycophenolate-mofetil-treated mice, and mice treated only with RT (62 vs. 42, 43, and 45.5 days, respectively; *p* < 0.05).

Overall, technological strides in preclinical RT have enhanced precision and delivery accuracy, overcoming initial limitations associated with whole-brain RT. Advanced RT platforms today achieve therapeutic radiation doses at the tumor site while sparing surrounding organs. Advanced RT techniques in GBM models with acquired RT resistance provide insights into resistance mechanisms and potential intervention targets. These advancements deepen our understanding and align with the clinical SOC, amplifying the translational potential for experimental therapies and promising improved outcomes in GBM.

### 4.3. Chemotherapy

TMZ is the chemotherapeutic drug in the GBM SOC and has been used in numerous preclinical studies. Research has largely focused on (A) characterizing the TMZ effect on immunotherapy [137,138,139], (B) combination studies to overcome TMZ resistance [140,141,142,143], and (C) identifying TMZ resistance mechanisms [144].

TMZ was first used in animal GBM models in 1994 [145]. In mice harboring U251 tumors, TMZ given via oral gavage at 600 mg/kg on day 1 or 200 mg/kg on days 1, 5, and 9 post-implantation increased the number of tumor-free mice on day 90 to 77.8% compared to only 10.5% in vehicle-treated mice. Similarly, in mice with SF-295 tumors, one 400 mg/kg dose or three 200 mg/kg doses increased the percentage of tumor-free mice at day 90 in comparison with vehicle controls (vehicle: 0%, 400 mg/kg: 30%, 200 mg/kg: 40%), and overall survival increased by 127%. In a meta-analysis of preclinical studies from 60 publications with a total of 2443 animals and 41 animal models, TMZ prolonged survival by 1.88-fold (95% CI 1.74–2.03) and reduced tumor volume by 54% (95% CI 41.8–58.9), with evidence of a dose–response effect for both outcomes [146]. Initiating TMZ treatment early (<20 days post-implantation) increased survival more than initiating treatment later (>20 days post-implantation), whereas later initiation decreased tumor volume more than early initiation. Local TMZ administration (intratumoral or intracerebral) increased survival to a greater degree than systemic administration (PO, IV, IP, or intragastric), but the reduction in tumor volume did not differ between local and systemic administration. TMZ pharmacokinetic parameters following IP, IV, and PO administration in various animal models are summarized in Table 2. Other dosing aspects, such as treatment duration and number of cycles, varied and were not considered in this meta-analysis [146]. Regarding model specificity, TMZ decreased tumor volume in human cell-based GBM models more than in rat and mouse cell-based GBM models, but median survival did not differ between human and animal models. Overall, this meta-analysis confirmed that TMZ is efficacious in preclinical models, but significant heterogeneities between studies and evidence of publication bias were pointed out.

#### 4.3.1. Route of Administration

While the route of TMZ administration varies across published preclinical studies, little research has been performed directly comparing administration routes. However, it becomes evident that the route of administration plays a significant role in TMZ delivery and efficacy, as demonstrated in a rat xenograft model of MGMT-negative lung cancer [153]. Animals were treated with a vehicle (saline) or either oral, intraventricular, or intraarterial TMZ (20 mg/kg). Quantitative autoradiography revealed that intraarterial administration significantly increased TMZ tumor concentrations three-fold compared to the normal brain, surpassing PO and IV administration (*p* < 0.02). Survival durations of rats dosed with the vehicle or PO, IV, or IA TMZ were 17.5, 25.5, 25.5, and 33 days, respectively. However, as shown by Evans blue extravasation, IA TMZ resulted in increased blood–brain barrier leakage, suggesting caution should be taken with IA TMZ administration in patients. These results mirror those seen in the meta-analysis by Hirst et al. [146], demonstrating superior efficacy of local vs. systemic TMZ administration.

#### 4.3.2. Dosing Schedule

The effect of altered dosing schedules on drug pharmacokinetics and pharmacodynamics has been studied more thoroughly than the administration route. Zhou et al. [154] compared metronomic (3.23 mg/kg/day for 28 days) and conventional dosing (18 mg/kg/day for 5 days) of TMZ in SF188V+ rat xenografts. Both schedules reduced the tumor volume, but metronomic dosing more effectively decreased VEGF and HIF-1α levels compared to conventional dosing at days 5, 14, and 28 post treatment initiation. In the slow-growing glioma model SVZ-EGFRwt, extending the time between TMZ doses to 7 and 13 days significantly increased survival compared to standard dosing (5 days/week, 10 mg/kg, i.p.) [155]. Delgado-Goñi et al. [156] used MRI and survival analyses to compare two different TMZ dosing schedules. GL261 mice received one cycle (60 mg/kg/day from days 11 to 15) or three cycles (60 mg/kg from days 11 to 15, 19 to 20, and 24 to 25). One TMZ cycle did not change survival compared to untreated mice; however, treatment with three cycles significantly decreased tumor growth and significantly increased median survival (33.8 days vs. 20.5 days; *p* = 0.0015).

In summary, integrating TMZ into preclinical GBM studies has been pivotal since its inception in 1994, increasing tumor-free survival percentages and prolonging overall survival of various GBM models. A comprehensive meta-analysis confirmed the effectiveness of TMZ, shedding light on the roles of regimen timing, varying administration routes, and model specificity. The impact of dosing schedules on pharmacokinetics and pharmacodynamics, explored through metronomic and conventional approaches, was demonstrated to produce variations in therapeutic outcomes.

While these findings contribute significantly to understanding the preclinical efficacy of TMZ, the existing heterogeneity across studies underscores the need for further standardization in experimental protocols. Achieving a consensus on dosing schedules, administration routes, and evaluation criteria will enhance the reliability and comparability of the results, ultimately advancing the translational potential of experimental therapies for GBM treatment.

### 4.4. Combination Therapies

In the remaining sections, we review combinations of the various SOC components in preclinical models, mirroring the comprehensive approach applied in clinical patient care.

#### 4.4.1. Resection + TMZ (or Other Novel Therapies)

Brain tumor resection in preclinical models allows for screening treatments that are applied to the resection cavity. Since there are limited preclinical studies with resection and TMZ, we summarize additional intracavitary therapies that are not part of the GBM SOC. In one of the earliest studies incorporating resection in an animal model, Akbar et al. [157] removed C6-GFP tumors from Wistar rats 15 days post-implantation, followed by direct administration of a TMZ-containing gel matrix in the resection cavity. After creating a 5 × 7 mm cranial window around the initial injection burr hole, the tumor was aspirated with a #3 F suction tip until no visible tumor tissue remained. Subsequently, the resection cavity was lined with a gel matrix containing varying TMZ concentrations (0%, 10%, 20%, or 30%). The dural defect was closed with DUREPAIR^®^, and the cranial window was reconstructed using TiMesh^®^. All groups showed a significant reduction in tumor burden, with the 30% TMZ group exhibiting a 94% average decrease in tumor weight compared to blank controls 15 days after resection and gel matrix administration. In a study by Graham-Gurys et al. [158], U87-mCherry-FLuc tumors were resected under fluorescence guidance, and resection cavities were lined with either acetylated dextran-doxorubicin or poly(L-lactide)-doxorubicin, significantly extending survival compared to untreated controls (*p* < 0.005 and *p* <0.05, respectively). Long-term survival at 120 days was higher for acetylated scaffolds than for poly(L-lactide) dextran (57% vs. 20%). Schiapparelli et al. [114] applied a similar technique with a camptothecin-based self-assembling prodrug hydrogel. Two weeks after implantation of GBM1A-GFP-Luc cells in mice, tumors were resected with a 3.0 mm punch biopsy tool, followed by debulking with microscissors. Applying camptothecin-containing hydrogel into the resection cavity significantly increased survival from 36 days to 64 days (*p* = 0.014) compared to mice that only underwent resection. At three weeks post-resection, mice receiving the prodrug hydrogel had 40% smaller tumors than those treated with control hydrogel. Wang et al. [115] also used punch biopsy to remove GL261 tumors, followed by intracavitary administration of a self-assembling paclitaxel hydrogel with or without an antibody against the macrophage immune checkpoint CD47. Treatment groups included saline, an empty filament control with/without anti-CD47 antibody, and a paclitaxel filament with/without anti-CD47 antibody. Mice treated with saline and the empty filament had similar median survivals (28.5 and 29.5 days). However, those receiving the empty filament with anti-CD47 antibody and paclitaxel alone showed significantly longer median survivals (39 and 63 days) and had 25% and 50% long-term survival at 80 days. Notably, paclitaxel, in combination with anti-CD47 antibody treatment, resulted in 100% survival.

Instead of using chemotherapeutic drugs, multiple groups have lined the resection cavities of preclinical models with therapeutic stem cells. Sheets et al. [117] outlined a procedure for this technique in the *Journal of Visualized Experiments*. The group resected U87-mCherry-FLuc tumors using a fluorescence dissecting stereomicroscope and lined the resection cavity with therapeutic stem cells in a poly(lactic acid) scaffold, demonstrating increased retention and anti-tumor cytotoxicity compared to direct injection; the survival of these mice was not measured. In a similar study, therapeutic stem-cell-seeded scaffolds were also implanted following the resection of U87-mCherry-FLuc tumors [116]. Stem cells were tagged with either a secretable luciferase (control) or tumor necrosis factor apoptosis-inducing ligand (treatment). While control mice had a median survival of 14.5 days post-resection, treatment mice showed a decrease in residual tumor cells by more than 80% at three days post-seeding, with 100% survival at six weeks post-resection. In the first preclinical study using this technique in combination with immune therapy, Choi et al. [159] resected CT2A-FmC tumors from immunocompetent C57BL/6 mice, observing reduced myeloid-derived suppressor cells and increased recruitment of CD4/CD8+ T cells 2–6 days post-surgery. Lining the resection cavity with IFNβ-secreting mesenchymal stem cells enhanced CD8+ T cell recruitment and increased survival in mice with CT2A-FmC and GBM4-FmC tumors. Similarly, Bhere et al. [160] implanted allogenic bifunctional mesenchymal stem cells into GBM-FmC resection cavities. The median survival of mice receiving no treatment and resection alone were 49 days and 91 days, respectively. Addition of the encapsulated stem cells into the resection cavity with subsequent injection of gancyclovir, a kill switch activator, resulted in 100% long-term survival (150 days).

Unlike studies that line the resection cavity with therapies, Datta et al. [161] developed a bihemispheric tumor model to assess tumor immune profiles before and after systemic treatment. In this model, mice were implanted with GL261, 005 GSC, or CT2A tumors. The same number of cancer cells were injected into both forebrain hemispheres, and tumor growth was monitored with 3D micro-ultrasound. When both tumors reached 2 mm in diameter, one tumor was resected and characterized, and then treatment with losartan w/wo anti-PD1 therapy was started. This approach revealed that, before the start of treatment, tumors from long-term-surviving mice were characterized by antitumor, immunostimulatory microenvironments, and with immune biomarkers correlated with survival. Taking all mice (responders and nonresponders) into account, GL261 and 005 GSC mouse survival doubled compared to anti-PD1 monotherapy with about 20% long-term survival. In contrast, no significant survival difference was observed across treatment groups in CT2A mice, which could be explained by the model’s excess extracellular matrix deposition and resistance to immune checkpoint inhibition.

Overall, incorporating resection into preclinical GBM models allows for exploring standard treatments and assessing additional intracavitary therapies beyond the clinical SOC. Various strategies involving the lining of resection cavities with TMZ or other therapeutic agents, such as drug-eluting hydrogels, immunomodulatory antibodies, or therapeutic stem cells, demonstrate the feasibility of such studies in small animals and showcase promising advancements in improving median survival and fostering long-term survivors. However, despite these encouraging findings, further standardization and comparative analyses are needed to successfully translate these preclinical strategies into effective clinical interventions for GBM patients.

#### 4.4.2. Radiation Therapy + TMZ

Differing dosing regimens for RT with TMZ impact on residual disease, and the effects of these treatments on the animals involved remain subject to active investigation. With an RT regimen already established in clinical practice [21], Lee et al. [162] compared conventional and pulsed low-dose RT in combination with TMZ in a preclinical mouse GBM model. U87 tumor-bearing mice received 14 Gy over 7 days, with the conventional dosing group receiving a daily continuous 2 Gy dose and the pulsed low-dose group receiving 10 pulses of 0.2 Gy with 3 min intervals. TMZ (10 mg/kg) was administered one hour prior to RT. Pulsed low-dose RT + TMZ resulted in longer median survival than conventional RT + TMZ (49 vs. 44 days; *p* = 0.09); however, both groups survived significantly longer than control animals (19 days; *p* < 0.001). McKelvey et al. [163] also studied two different RT dosing regimens, with mice receiving either conventional (20 Gy/10 fractions) or hypofractionated (20 Gy/4 fractions; 72 h intervals) RT using the Small Animal Radiotherapy Research Platform. Hypofractionated RT improved median survival compared to conventional RT in GL261 and CT2A models (GL261: 74.5 vs. 33 days, *p* < 0.0001; CT2A: 100 vs. 44.5 days, *p* < 0.0001). However, when combined with TMZ, neither regimen reached median survival by day 100. To assess the radiosensitizing effect of TMZ on conventional RT, Carlson et al. [164] treated a panel of 20 mice with patient-derived GBM xenografts model using TMZ (66 mg/kg × 5 day), RT (2 Gy 2×/day × 5 day), TMZ/RT, or a placebo. Combined therapy yielded additional survival benefits only in a subset (45%) of *MGMT* methylated tumors, with no such benefit observed in *MGMT* unmethylated tumors. Furthermore, the sequence of treatment mattered, as RT followed by TMZ was less efficacious than TMZ followed by RT or concurrent treatment (survival ratios: 4.0, 9.6, and 12.9, respectively; *p* < 0.0001), suggesting a true radiosensitizing effect of TMZ on RT.

Since recurrence is still nearly ubiquitous following RT + TMZ, Palanichamy et al. [165] sought to characterize the effect of concurrent RT and TMZ on residual GBM cells in a panel of models developed with patient-derived and commercially available cell lines. Mice received five cycles of the following treatment—day 1: TMZ (100 mg/kg), day 2: RT (1 Gy), day 3: monitoring. Concurrent RT/TMZ enriched a population of cells with increased induced pluripotent stem cell gene expression and enhanced tumorigenicity. Cameron et al. [166] had similar findings, showing that neural stem cells survive concurrent RT/TMZ and adjuvant TMZ therapy due to increased anti-apoptotic protein Bcl2 and Mcl1 expression.

Transitioning from the cellular level to animal behavior, Dey et al. [167] irradiated C57BL/6 wild-type mice with three fractionated X-ray doses (8.67 Gy every other day) and concurrent TMZ (25 mg/kg), followed by nine additional adjuvant TMZ doses (67 mg/kg) over three weeks. Control mice received sham radiation and vehicle injections. To eliminate the neurological effects of GBM itself, mice did not have tumors. Mice treated with RT/TMZ demonstrated anxiety-like behaviors at 5 weeks post-treatment and both anxiety- and depression-like symptoms at 15-weeks post-treatment, with a 50% decrease in hippocampal 5HT1A serotonin receptor levels and a 37% decrease in serotonin levels. Fluoxetine, a serotonin reuptake inhibitor, reversed depression-like behavior. This highlights the importance of similar studies to improve the overall quality of life for GBM patients.

The combination of RT and TMZ in preclinical models has provided valuable insights into dosing regimens, their impact on residual disease, and effects on animal behavior. The studies outlined above highlight the potential benefits of combined therapy, demonstrating prolonged survival in diverse model systems. Notably, variations in dosing sequences and the molecular characteristics of tumors influence treatment efficacy. However, from the studies in the section above, it is evident that preclinical research using RT and TMZ is not standardized, with doses ranging widely across studies. Overall, these findings underscore the significance of comprehensive preclinical investigations, including components of the SOC, to enhance the overall therapeutic outcomes for individuals with GBM.

#### 4.4.3. Radiation Therapy + TMZ + Novel Therapeutic Strategies

Despite combining RT and TMZ, GBM patient survival is still dismal. Therefore, preclinical studies test RT/TMZ–drug combinations for additive or synergistic effects to further improve patient outcomes. The compounds tested have a variety of mechanisms of action ranging from enzyme inhibitors to immune-based strategies. We discuss these strategies below in chronological order.

Chaponis et al. [168] studied the combination of RT (2.5 Gy/day for 2 days), TMZ (5 mg/kg 90 min before RT), and the farnesyl transferase inhibitor lonafarnib (80 mg/kg q.d.) in U87-bearing mice. Lonafarnib alone did not reduce the tumor burden, but lonafarnib with RT/TMZ decreased the tumor burden by about 3-fold by week four compared to the control (*p* < 0.05). In a study using a genetically engineered mouse GBM model, mice were treated alone or in a combination of RT, TMZ, and the PARP inhibitor ABT-888 [169]. TMZ and ABT-888 were given concomitantly and as maintenance therapies relative to RT, to mirror the current SOC. TMZ/RT/ABT-888 mice had a longer time-to-progression, progression-free survival, and median survival than all other treatment groups. Specifically, the time-to-progression and median survival for mice in the TMZ/RT and TMZ/RT/ABT-888 groups were 20 vs. 35 days and 25 vs. 36.5 days, respectively. Festuccia et al. [170] explored using the alkylating deacetylase inhibitor tinostamustine in combination with RT. Since invasive tumor cells remain in or around the tumor bed following SOC treatment, the authors inoculated a low number of tumor cells (3000 cells) as a surrogate for recurrence. TMZ or tinostamustine was given alone or in combination with RT starting 5 days post-inoculation. In both U251 and GSC-5 GBM mice, tinostamustine + RT had a greater median survival compared to TMZ + RT (U251: ~140 vs. 170 days; GSC-5: ~150 vs. 200 days), suggesting that tinostamustine is a stronger radiosensitizer than TMZ. Lastly, Burgenske et al. [171] used an orthotopic patient-derived xenograft mouse GBM model panel to assess the efficacy of the tumor checkpoint controller lisavanbulin w/wo RT/TMZ. In a study using a complete chemo-RT regimen, mice bearing GBM39, GBM150, or GBM26 tumors received two weeks of RT (2 Gy × 10 fractions) with concurrent TMZ (20 mg/kg) followed by three cycles of adjuvant TMZ (50 mg/kg; days 1–5 every 28 days) w/wo daily lisavanbulin (30 mg/kg). For mice with GBM150 tumors, adding lisavanbulin did not extend survival compared to RT/TMZ (98 vs. 123 days; *p* = 0.97). However, lisavanbulin significantly increased survival in the other two models (GBM39: 502 days vs. 249 days, *p* = 0.0001; GBM26: 172 days vs. 121 days, *p* = 0.04).

In conclusion, research on novel therapeutic strategies in preclinical models emphasizes the pivotal role of combining RT and TMZ. Various studies, spanning different therapeutic approaches, demonstrate the enhanced efficacy of these agents when combined with RT/TMZ. These findings provide valuable insights for developing more effective and comprehensive treatment strategies for GBM compared to the current clinical SOC, emphasizing the continued relevance of RT/TMZ treatment in advancing novel therapeutic interventions. A significant limitation of these studies is the absence of the third component of the SOC, surgery, which also influences the GBM microenvironment and impacts the effectiveness of combination therapies.

#### 4.4.4. Resection + Radiation Therapy + Chemotherapy (Stupp Protocol)

**Figure 3:**
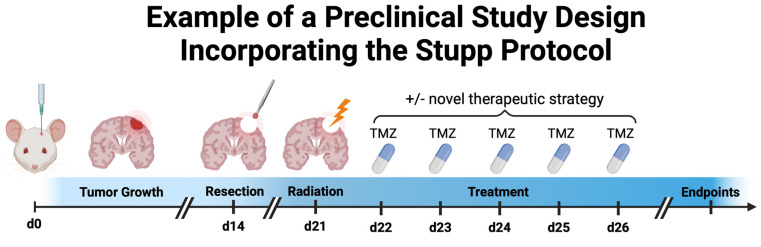
Example of a preclinical study design incorporating the Stupp protocol. In a preclinical study following the Stupp protocol, mice receive intracranial tumor implants on day 0. After allowing sufficient time for tumor growth and confirmation of successful engraftment using in vivo imaging techniques like MRI or bioluminescence imaging, tumors are resected. Subsequently, mice undergo radiation therapy using a small animal radiation platform and chemotherapy with temozolomide. Novel therapeutic strategies are combined with or compared to the Stupp protocol to evaluate their effects on primary endpoints. Adapted from “Mouse Experimental Timeline”, by BioRender.com (2024). Retrieved from https://app.biorender.com/biorender-templates/figures/all (accessed on 21 February 2024).

To fill the abovementioned gap, some groups have recapitulated the full Stupp protocol in preclinical models by combining novel treatment options with resection, radiation, and chemotherapy. An example of a preclinical study incorporating the SOC is depicted in Figure 3.

Reste et al. [172] resected tumor tissue from GL261 GBM mice using intravenous fluorescein and filled the resection cavity with the IRE1 inhibitor MKC8866. Three days after the resection, the authors completed a treatment plan similar to the Stupp protocol consisting of RT (5 × 2 Gy) and chemotherapy (5 × 25 mg/kg TMZ), followed by 30–50 mg/kg TMZ for 5 days with 2–3 days between each treatment. Resection alone did not change median survival compared to control animals, but treatment with the Stupp protocol doubled median survival. Dr. Rakesh Jain’s group conducted multiple studies treating mice with the SOC alone and in combination with anti-PD1 therapy. In the first study, CT2A-GFP-GLUC tumors were resected after reaching 5–10 mm^3^ as determined by GLUC activity, followed by SOC treatments [RT (1 Gy/day); TMZ (25 mg/day)] for 10 days [173]. RT/TMZ significantly increased survival from 8 days (IgG control) to 14 days post-resection (*p* < 0.001). Anti-GITR and anti-PD1 antibodies extended survival to 42 days (*p* < 0.001). In another study, Datta et al. [161] studied the combination of losartan with the SOC and anti-PD1 therapy in mice implanted with GL261 cells. When tumors reached 2 mm in diameter, they were removed. RT (2 Gy/day for 5 days) and TMZ (25 mg/kg for 10 days) were initiated two days post-resection. While losartan + SOC did not significantly change the median survival compared to SOC alone (~40 days), SOC + anti-PD1 therapy and SOC + losartan + anti-PD1 led to 17% and 43% long-term survival (no detectable tumor at day 80).

Riva et al. [7] studied the clinical SOC in CT-2A tumor-bearing mice. Fluorescence-guided resection was performed on day 14 post-implantation, followed by focal RT (4 Gy) and four doses of TMZ (50 mg/kg) on alternate days starting on day 28. Compared to untreated control mice (median survival 34 days), 63% of mice that underwent resection, RT, and chemotherapy were still alive by the end of the study (day 75).

In summary, several studies have aimed to replicate the Stupp protocol in preclinical GBM models by combining various treatment options with resection, radiation, and chemotherapy. For instance, combining fluorescein-guided resection with IRE1 inhibitor treatment, radiation, and chemotherapy doubled median survival compared to resection alone. Additionally, other studies investigated the efficacy of SOC treatment alone or in combination with immunotherapy, demonstrating significant improvements in survival. Despite treatments mirroring the clinical SOC, their higher efficacy in mice compared to patients highlights the need for further optimization to align with clinical outcomes.

## 5. Conclusions and Future Directions

Since 2005, the SOC for newly diagnosed GBM patients has been surgery, RT, and chemotherapy with TMZ. Nevertheless, overall patient survival outcomes remain grim. Our understanding of the molecular GBM pathogenesis and the development of novel treatment strategies has increased within the past few decades, but translation of research from the workbench to the bedside is not always successful, and this step is one obstacle to developing new treatments and improving patient survival.

Increasing evidence supports the notion that each component of the GBM SOC alters the remaining disease course, including effects on tumor growth characteristics and the tumor microenvironment, emphasizing the difference between treatment-naïve and recurrent tumors. The effects of surgery, RT, and chemotherapy hold true in animal models, emphasizing that it is important to mirror the clinical SOC in preclinical studies and increase the translational potential of novel therapeutic strategies.

The advent of small animal RT platforms and microsurgical techniques allows for recreating the entire SOC in animal GBM models, and this review shows the importance of incorporating the SOC as a suitable control in preclinical studies. However, from the studies compiled in this review, it is also evident that surgical techniques, RT dosing regimens, and TMZ doses and routes of administration are not standardized, making it difficult to compare studies or determine which strategies are optimal for clinical translation. Thus, we recommend that future investigations prioritize establishing a standardized preclinical SOC replicating GBM patient care’s intricacies.

A limitation of many preclinical studies discussed in this review is the use of concurrent or immediately sequential combination therapy (e.g., SOC + new agent). This approach can potentially prevent tumor recurrence and improve initial treatment efficacy. However, this strategy does not fully address the genetic and phenotypic evolution that occurs in recurrent tumors, which may have different vulnerabilities compared to primary tumors. True recurrence models involve treating the primary tumor with the SOC, allowing the tumor to recur, and then testing new therapies on the recurrent tumor. These models better mimic the clinical scenario and provide insights into the treatment-resistant characteristics of recurrent tumors. However, true recurrence models are time-consuming and resource-intensive, which can limit their widespread use. Nonetheless, in clinical practice, the standard of care is completed sequentially (resection, followed by RT and chemotherapy), making this approach potentially more appropriate for preclinical study design. Future research should explore the impact and significance of the timing when integrating these standard treatments with new therapies.

Animal models cannot fully recapitulate the complexity of human GBM, but incorporating the SOC into preclinical research holds the promise of improving the translation of experimental therapies into clinical practice with a therapeutic benefit for GBM patients.

## Figures and Tables

**Table 1:** Examples of targeted imaging agents.

Imaging Agent	Target	Tumor:Normal Tissue Ratio
IRDye 800CW-RGD	Integrin receptor	16–18:1
Anti-TRP-1-Alexa fluor 488 or 750	TRP	unspecified
Anti-EGFR antibodies	EGFR	200–1000:1
Angiopep-2-Cy5.5	LRP	1.6:1

**Table 2:** Plasma, brain, and CSF pharmacokinetic parameters of temozolomide in preclinical models.

Source	Animal	Sex	Route	Dose	t_1/2_	T_max_	C_max_	AUC **
				[mg/kg]	[h]	[h]	[µg/mL]	[h × µg/mL]
**Plasma**								
Goldwirt et al., 2013 [147]	Swiss mice	F	IP	66	0.88	0.25	27.89	31
de Gooijer et al., 2018 [148]	FVB mice	M	IV	50	0.69		~55	65.4
Zhang et al., 2021 [149]	ICR mice	M	PO	30	4.04	0.25	20.96	39.9
Reyderman et al., 2004 [150]	Sprague–Dawley rats	M	IV	16.6 *	1.2	0.08	34.7	55.6
Reyderman et al., 2004 [150]	Sprague–Dawley rats	F	IV	16.6 *	1.1	0.08	35.3	50.6
Reyderman et al., 2004 [150]	Sprague–Dawley rats	M	PO	16.6 *	1.17	0.75	21.5	55.2
Reyderman et al., 2004 [150]	Sprague–Dawley rats	F	PO	16.6 *	1.22	0.25	31.4	56.4
Reyderman et al., 2004 [150]	Long–Evans rats	M	PO	16.6 *	1.4	0.25	27.5	78.8
Reyderman et al., 2004 [150]	Long–Evans rats	F	PO	16.6 *	1.2	0.25	40.9	91.6
Patel et al., 2003 [151]	Rhesus monkey	M	IV	7.5	1.5	1.5	20.19	76.1
**Brain**								
Goldwirt et al., 2013 [147]	Swiss mice	F	IP	66	0.91	0.75	6.63	8.5
de Gooijer et al., 2018 [148]	FVB mice	M	IV	50			~23	36.8
Zhang et al., 2021 [149]	ICR mice	M	PO	30	4.2	0.5	20.62	53.3
Reyderman et al., 2004 [150]	Sprague–Dawley rats	M	IV	16.6 *	1.2	0.25	11.4	21.8
Reyderman et al., 2004 [150]	Sprague–Dawley rats	F	IV	16.6 *	1.1	0.08	11.1	18.5
Reyderman et al., 2004 [150]	Sprague–Dawley rats	M	PO	16.6 *	1.3	1	7.9	19.8
Reyderman et al., 2004 [150]	Sprague–Dawley rats	F	PO	16.6 *	1.1	0.5	8.3	17.5
**CSF**								
Patel et al., 2003 [151]	Rhesus monkey	M	IV	7.5	1.5	2.5	5.05	24.5

* The dose in rats was converted from mg/m^2^ to an equivalent dose in mg/kg by dividing by 12 [152]. ** For mice, the AUCs were calculated to the last time point collected (AUC_last_); for rats and monkeys, the AUCs were calculated to infinity (AUC_inf_).
